
JOURNAL INFORMATION
==============================
NLM Title Abbreviation: Biospektrum (Heidelb)
Journal ID: Biospektrum (Heidelb)
NLM Journal ID: 9615685

Biospektrum

ISSN: 0947-0867
EISSN: 1868-6249

ARTICLE INFORMATION
==============================
PMCID: PMC8611386
DOI: 10.1007/s12268-021-1656-7
Article ID: 1656
Article version: 1
Subjects: Karriere, Köpfe & Konzepte

Buchrezension zu: Die Idee des Humanen

Die Idee des Humanen Rudolf Virchow und Hermann von Helmholtz Das Erbe der Charité, Ernst Peter Fischer und Detlev Ganten, 263 S, Hirzel, 2021. HC, 22,90 €. ISBN: 9783777629025, Auch als E-Book erhältlich

Seifert Roland seifert.roland@mh-hannover.de

grid.10423.34 0000 0000 9529 9877 Medizinische Hochschule, Hannover, Deutschland
Electronic publication date: 2021 Nov 24
Print publication date: 2021
Volume: 27
Issue: 7
First page: 786
Last page: 786
Copyright: © Der Autor 2021
License: Dieser Artikel wird unter der Creative Commons Namensnennung 4.0 International Lizenz veröffentlicht, welche die Nutzung, Vervielfältigung, Bearbeitung, Verbreitung und Wiedergabe in jeglichem Medium und Format erlaubt, sofern Sie den/die ursprünglichen Autor(en) und die Quelle ordnungsgemäß nennen, einen Link zur Creative Commons Lizenz beifügen und angeben, ob Änderungen vorgenommen wurden. Die in diesem Artikel enthaltenen Bilder und sonstiges Drittmaterial unterliegen ebenfalls der genannten Creative Commons Lizenz, sofern sich aus der Abbildungslegende nichts anderes ergibt. Sofern das betreffende Material nicht unter der genannten Creative Commons Lizenz steht und die betreffende Handlung nicht nach gesetzlichen Vorschriften erlaubt ist, ist für die oben aufgeführten Weiterverwendungen des Materials die Einwilligung des jeweiligen Rechteinhabers einzuholen. Weitere Details zur Lizenz entnehmen Sie bitte der Lizenzinformation auf http://creativecommons.org/licenses/by/4.0/deed.de.
License URL: https://creativecommons.org/licenses/by/4.0/

issue-copyright-statement: © Springer-Verlag GmbH Deutschland, ein Teil von Springer Nature 2021

==============================
Funding note

Open Access funding enabled and organized by Projekt DEAL.

Diese Rezension erscheint Open Access.

